# Identifying a survival-associated cell type based on multi-level transcriptome analysis in idiopathic pulmonary fibrosis

**DOI:** 10.1186/s12931-024-02738-w

**Published:** 2024-03-15

**Authors:** Fei Xu, Yun Tong, Wenjun Yang, Yiyang Cai, Meini Yu, Lei Liu, Qingkang Meng

**Affiliations:** Department of Pharmacogenomics, College of Bioinformatics Science and Technology, Harbin Medical University, Harbin, 150081 China

## Abstract

**Background:**

Idiopathic pulmonary fibrosis (IPF) is a progressive disease with a five-year survival rate of less than 40%. There is significant variability in survival time among IPF patients, but the underlying mechanisms for this are not clear yet.

**Methods and results:**

We collected single-cell RNA sequence data of 13,223 epithelial cells taken from 32 IPF patients and bulk RNA sequence data from 456 IPF patients in GEO. Based on unsupervised clustering analysis at the single-cell level and deconvolution algorithm at bulk RNA sequence data, we discovered a special alveolar type 2 cell subtype characterized by high expression of CCL20 (referred to as ATII-CCL20), and found that IPF patients with a higher proportion of ATII-CCL20 had worse prognoses. Furthermore, we uncovered the upregulation of immune cell infiltration and metabolic functions in IPF patients with a higher proportion of ATII-CCL20. Finally, the comprehensive decision tree and nomogram were constructed to optimize the risk stratification of IPF patients and provide a reference for accurate prognosis evaluation.

**Conclusions:**

Our study by integrating single-cell and bulk RNA sequence data from IPF patients identified a special subtype of ATII cells, ATII-CCL20, which was found to be a risk cell subtype associated with poor prognosis in IPF patients. More importantly, the ATII-CCL20 cell subtype was linked with metabolic functions and immune infiltration.

**Supplementary Information:**

The online version contains supplementary material available at 10.1186/s12931-024-02738-w.

## Introduction

Idiopathic pulmonary fibrosis (IPF) is a severe chronic interstitial lung disease [1]. IPF patients have a poor prognosis, with most patients dying within 2–3 years after diagnosis [2, 3] and a survival rate of less than 40% at 5 years [4, 5]. IPF patients experience the destruction of alveolar structures, resulting in decreased lung function, interrupted gas exchange, respiratory failure, and ultimately death [6]. Despite increasing research on IPF [7–11], the factors that impact the prognosis of IPF patients remain unclear. Currently, only two drugs, Nintedanib and Pirfenidone are used to slow down the progression of IPF [12, 13], however, the administration of these two drugs is standardized, with little consideration given to the severity of the disease and individual molecular, genetic, and genomic variations [14]. It was known that the clinical progression of IPF patients was heterogeneous, with some progressing rapidly leading to poor prognosis and early death, while others showed very little deterioration and better prognosis [15, 16]. The reasons for these differences in IPF progression were not yet clear. Therefore, it is imperative to identify effective biomarkers for early identification of IPF patients with poor prognoses.

Pulmonary epithelial cells play a critical role in the pathogenesis of IPF. In IPF patients, the epithelial cells undergo phenotypic and functional changes, which “reprogram” their normal repair response to injury, involving fibroblast activation, extracellular matrix remodeling, ultimately leading to fibrosis [17]. These changes in pulmonary epithelial cells can lead to permanent scar formation and organ dysfunction, ultimately resulting in premature death. Recently, single-cell RNA sequencing (scRNA-seq) technology has been used to capture the RNA of individual cells and sequence it, providing a finer resolution to describe the transcriptional heterogeneity of cell populations in IPF as well as the biological processes and pathogenesis associated with IPF [18–21]. scRNA-seq can discover new cell types and molecular mechanisms, and reveal cell heterogeneity. Bulk RNA sequencing (bulk RNA-seq) technology can reveal the overall characteristics and average gene expression levels of many patient tissues. Therefore, combining bulk RNA-seq data from a large number of patients with extensive scRNA-seq data can provide a clearer understanding of the dynamic process of IPF development, more accurate identification of biological factors affecting the prognosis of IPF patients, and ultimately promote the development of clinical treatments.

Clinical and experimental evidence indicated that IPF represented an epithelial-driven disorder [22–25]. So, we hypothesized that there may be unknown epithelial cell subtypes that can affect survival as the cell proportion increases in IPF patients. To address this, unsupervised clustering analysis of epithelial cells from IPF patients was first performed based on scRNA-seq data, followed by deconvolution analysis of IPF bulk RNA-seq data based on the unsupervised clustering results to reveal the impact of epithelial cell subtype proportion on the survival time of IPF patients. Finally, we discovered a special subtype of ATII cells, named ATII-CCL20, which is characterized by abnormal expression of metabolic and immune-related genes. Many previous studies have analyzed epithelial cells in IPF [26–28], but they didn’t identify the epithelial cell subtype associated with survival. In contrast, our study has discovered a new subtype of epithelial cells, ATII-CCL20, IPF patients with a higher proportion of ATII-CCL20 have significantly worse survival outcomes in multiple datasets. In summary, our study reveals a risk cell subtype ATII-CCL20 and elucidates its functional changes in IPF. More importantly, we generated a comprehensive decision tree and nomogram model to optimize the risk stratification of IPF patients, providing a reference for accurate prognosis evaluation of IPF patients and a new direction for treatment.

## Materials and methods

### Data processing of single-cell RNA-sequencing (scRNA-seq) data

To investigate the heterogeneity of epithelial cells from IPF patients, we collected two scRNA-seq datasets GSE136831 [20] and GSE135893 [29] in the GEO [30], while GSE136831 was utilized as the discovery dataset, GSE135893 served as the validation dataset. Raw UMI count data was used and epithelial cells were extracted for subsequent analysis. The R package Seurat [31] was extensively utilized for the systematic processing of scRNA-seq data in this study. First, we removed the genes that were not expressed in all cells. The scRNA-seq expression profiles were log-normalized using the NormalizeData function. To improve the accuracy of downstream unsupervised clustering analyses, we performed highly variable gene selection using the FindVariableFeatures function based on the mean.var.plot (MVP) method and 966 high variant genes were identified from 18,088 genes. Next, the R function “ScaleData” was used to scale the expression of highly variable genes to balance the weight of genes in the downstream analysis. The RunPCA function was performed to principal component analysis (PCA) [32] on the highly variable genes and selected the optimal number of principal components (PC) using a combination of the JackStraw method and Elbow method. 45 PCs were selected, then, we used the FindClusters function to perform unsupervised clustering of the epithelial cells with a resolution of 1. The clustering results were visualized using the t-distributed stochastic neighbor embedding (tSNE) method [33]. The FindAllMarkers function was used to identify marker genes for each epithelial cell subtype, with a significance threshold set at |avg_log2FC|> 2 and p_val_adj < 1e-2. The DimPlot and VlnPlot functions were used to visualize marker gene expression and distribution.

### Data processing of bulk RNA-sequencing (bulk RNA-seq) data

Two datasets of peripheral blood mononuclear cell (PBMC) from IPF patients were selected from the GEO database, GSE27957 [34] and GSE28042 [34], as well as one dataset of bronchoalveolar lavage fluid (BALF) from IPF patients, GSE70866 [35]. For data preprocessing, the ComBat function from the R package sva [36] was used to remove the batch effect between the three datasets. The clinical characteristics of patients in each dataset were shown in Additional file 1: Table S1. The lung tissue of 160 IPF samples were selected from GSE47460 [37].

### Identification of risk cell subtype

To expand the epithelial cell subtypes composition analysis to a larger number of IPF patients, we performed deconvolution analysis employing CIBERSORTx [38] on the aforementioned four bulk RNA-seq datasets, setting the perm to “1000” and QN to “TRUE”. The feature matrix was constructed from the expression profile of DEGs of each epithelial cell subtype.

To investigate the effect of epithelial cell subtypes on the survival outcomes of IPF patients, we performed the associations between survival outcomes and epithelial cell subtypes estimates. The associations were carried out with the surv_cutpoint function from the R package survminer [39] to determine the optimal cutoff values for cell subtype proportion in IPF patient grouping in GSE70866, GSE27957, and GSE28042 datasets. Kaplan–Meier analysis was performed using the R package survival [40] to compare the differences in survival time between IPF patient groups.

### Biological characteristics analysis of ATII-CCL20 cell subtype in single-cell data

Through the prognostic analysis, we found that the proportion of the ATII-CCL20 cell subtype significantly affected the prognosis of IPF patients. To understand the evolutionary relationship between the ATII-CCL20 cell subtype and other cell subtypes, we performed a single-cell pseudo-time analysis using the R package Monocle [41]. Logarithmically normalized data was used as input and DEGs for cell subtypes were used as the ordering genes. The learn_graph function and order_cells function were used for cell trajectory inference, with both using default parameters. To analyze the metabolic functional differences between the ATII-CCL20 cell subtype and other epithelial cell subtypes, the R package scMetabolism [42] was used to evaluate single-cell metabolic activity with the “VISION” method and KEGG pathways [43] built into scMetabolism as input. To analyze the interaction between the ATII-CCL20 cell subtype and other epithelial cell subtypes, the R package NicheNet [44] was used to perform the cell communication, using common databases (KEGG, ENCODE, PhoshoSite) to track downstream effectors such as transcription factors and receptor targets in the provided dataset. The predict_ligand_activities function was used to calculate the expression levels of ligands in each cell subtype, and the prepare_ligand_target_visualization function was used to visualize the strength of interactions between receptors and ligands.

To understand the functional differences between ATII-CCL20 and other ATII cell subtypes deeply, we used the FindMarkers function in the R package Seurat [31] to identify DEGs between ATII-CCL20 and other ATII cell subtypes, with a threshold set at |avg_log2FC|> 2 and p_val_adj < 1e-2. The R package clusterProfiler [45] was employed to perform enrichment analysis on DEGs, with a threshold set at adj p < 0.05.

### Functional analysis of a high proportion of ATII-CCL20 IPF patients in bulk RNA-seq data

In order to further reveal the biological functions related to the ATII-CCL20 cell subtype using bulk RNA-seq data, we divided all IPF patients from GSE27957, GSE28042, and GSE70866 datasets based on the proportion of ATII-CCL20 into two groups (high-ATII-CCL20 and low-ATII-CCL20). The R package limma [46] was used to identify the DEGs between high ATII-CCL20 and low ATII-CCL20 IPF patients, with a threshold set at |logFold Change(logFC)|> 1 and adj p < 0.05. Additionally, the R package clusterProfiler was used to conduct functional enrichment analysis on DEGs, with a threshold set at adj p < 0.05. Simultaneously, the proportion of ATII-CCL20 in the GSE47460 dataset was sorted, with the top 30% in terms of high proportion selected as the high ATII-CCL20 group, and the top 30% in terms of low proportion selected as the low ATII-CCL20 group. To determine potential signaling pathways associated with ATII-CCL20 proportion, we collected 50 hallmark pathways (h.all.v7.1.symbols) in the Molecular Signatures Database (MSigDB) [47], using the R package GSVA [48] to perform gene set variation analysis (GSVA) on high ATII-CCL20 and low ATII-CCL20 IPF patients, and got the GSVA enrichment scores for 50 hallmark pathways [47–49]. Kaplan–Meier analysis was further performed to demonstrate the prognostic impact of the 50 hallmark pathways. Venn diagram analysis was performed to determine overlapping hallmark pathways in GSVA and Kaplan–Meier analysis. To study the infiltration of immune cells in high ATII-CCL20 and low ATII-CCL20 IPF patients, we implemented the ssGSEA algorithm [50] to quantify the relative infiltration of 28 reported immune cell types [51], box plot showed the results. Pearson correlation coefficient (PCC) was calculated to determine the correlation between the 28 immune cells in high ATII-CCL20 and low ATII-CCL20 IPF patients, respectively.

### Construction of a predictive nomogram

Univariate Cox regression and multivariate Cox regression were used to estimate the hazard ratio (HR) of cell subtypes proportion and other clinical indicators. A clinical prediction nomogram was constructed using the R package rms [52]. To quantify the performance of the nomogram, a calibration curve was generated by comparing the predicted values of the nomogram with the observed actual survival rates. The calibration curve was used to evaluate the consistency between our predicted values and the reality. Decision curve analysis (DCA) was performed using the R package ggDCA [53], and a clinical impact curve was plotted to evaluate whether nomogram-based decisions were beneficial for IPF patient prognosis.

## Result

### The epithelial landscape of IPF patients revealed by scRNA-seq analysis

**Fig. 1:**
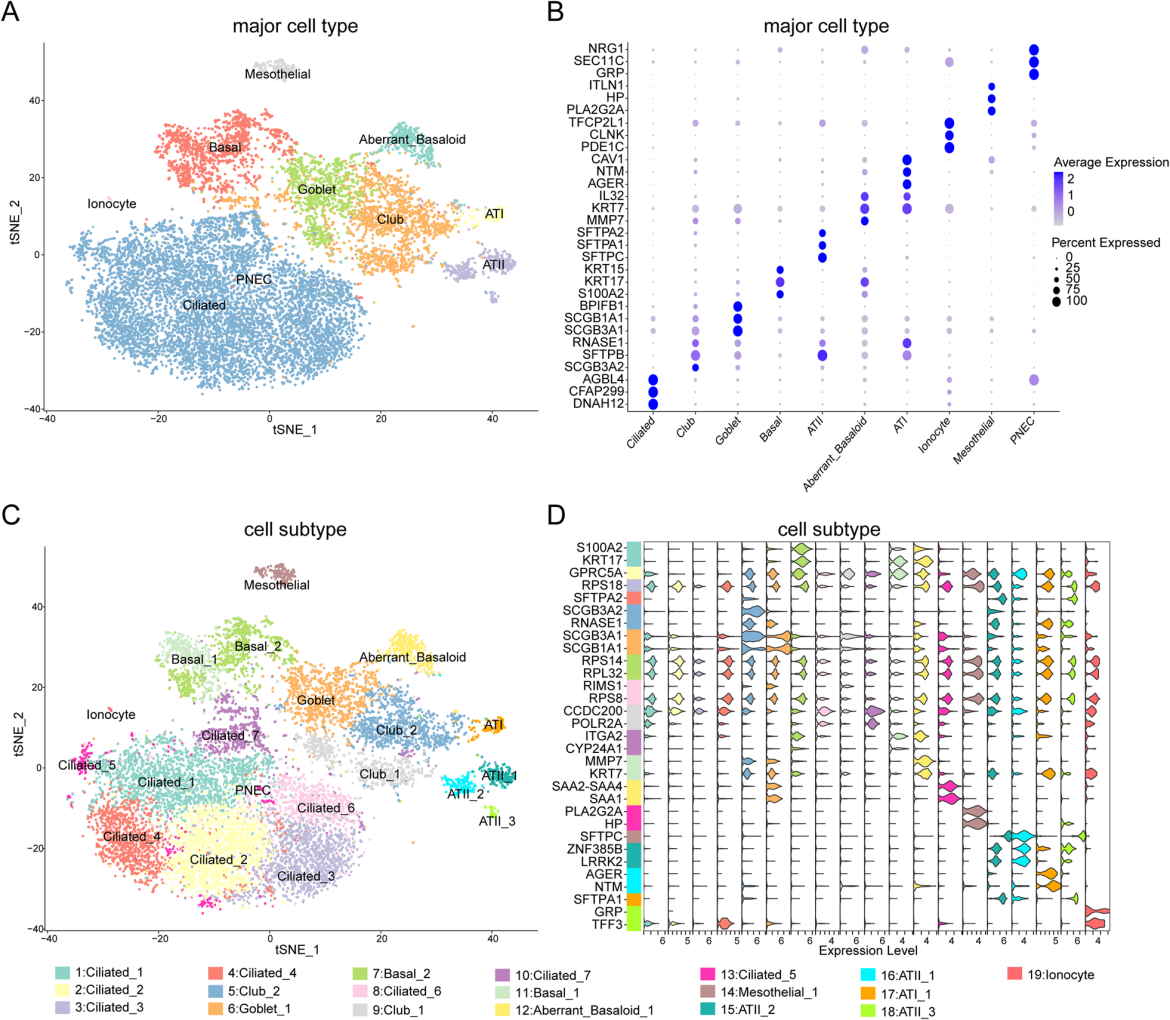
Analysis of the epithelial cell landscape in 32 IPF patients using scRNA-seq data in GSE136831. **A**, **B** tSNE plots of 10 major epithelial cell types and dot plots of their corresponding marker genes. **C**, **D** tSNE plots of 19 cell subtypes and the violin plots of their corresponding marker genes

The overall study design was displayed in Additional file 1: Figure S1. To systematically reveal the characteristics of IPF epithelial cells, we collected 13,223 epithelial cells from 32 IPF patients in GSE136831 and employed the R package Seurat to process the scRNA-seq data and perform unsupervised clustering. 13,223 epithelial cells were classified into 10 major cell types: aberrant basaloid cells, alveolar type 1 (AT1) cells, alveolar type 2 (AT2) cells, basal cells, goblet cells, ionocytes, mesothelial cells, pulmonary neuroendocrine cells (PNEC), ciliated cells, and club cells (Fig. 1A). Differential analysis was performed on the 10 major cell types and 7714 marker genes were identified (adj p < 0.05, Fig. 1B). Among them, ATI cells highly expressed typical marker AGER, while Goblet significantly upregulated SCGB3A1, SCGB1A1, and BPIFB1 (Fig. 1B). At the same time, 13,223 epithelial cells were clustered into 19 independent cell subtypes, and ATII cells were divided into 3 cell subtypes, ciliated cells into 7 cell subtypes, basal cells into 2 cell subtypes, and club cells into 2 cell subtypes (Fig. 1C). Meanwhile, differential analysis was performed on the 19 cell subtypes and 8442 marker genes were identified (adj p < 0.05, Fig. 1D). We found that there were multiple cell subtypes in a major cell type, but the marker genes among these cell subtypes were different. Meanwhile, we found that there were differences in the composition of 10 major epithelial cell types or 19 independent cell subtypes in IPF patients (Additional file 1: Figure S2), which indicated that the epithelial cells of IPF patients exhibited obvious heterogeneity, which might be the primary reason for the significant clinical differences observed in IPF patients.

### The ATII-CCL20 cell subtype is associated with IPF patients’ survival outcomes

**Fig. 2:**
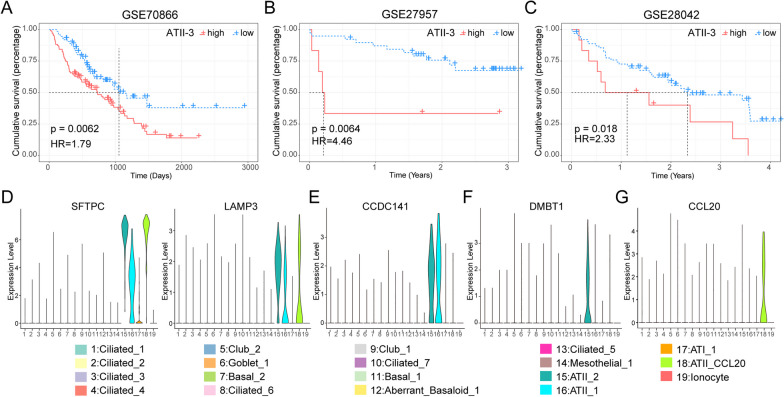
The correlation between the increased proportion of ATII-CCL20 cell subtype and poor prognosis in IPF patients. **A**–**C** The Kaplan–Meier survival curves for overall survival (OS) of IPF patients in GSE70866 (**A**), GSE27957 (**B**), and GSE28042 (**C**) showed worse survival outcomes for IPF patients with a high proportion of ATII-CCL20 cell subtype. **D** Typical marker genes of ATII cell: SFTPC and LAMP3. **E**, **F** Marker genes of ATII-1 and ATII-2 cell subtype. **G** Marker genes of ATII-CCL20 cell subtype. **H** ROC curve of the binary classification model for the proportion of ATII-CCL20 cell subtype in the training set GSE70866. **I**, **J** ROC curves of the binary classification model for the proportion of ATII-CCL20 cell subtype in the validation datasets GSE27957 (**I**) and GSE28042 (**J**)

Due to the significant differences in prognosis among IPF patients and the large heterogeneity of epithelial cells observed in previous analyses, we hypothesized that there might be one or more epithelial cell subtypes associated with IPF patient prognosis. To determine the clinical significance of epithelial cell subtypes, first, we performed deconvolution analysis using CIBERSORTx on the bulk RNA-seq datasets and got the proportion of 19 epithelial cell subtypes in IPF patients. It was found that there were significant differences in the proportion of 19 cell subtypes, which confirmed the heterogeneity in IPF patients again. Then we correlated the proportion of the 19 epithelial cell subtypes with the survival outcomes of 176 IPF patients in GSE70866. Interestingly, the result showed that a high proportion of the ATII-3 cell subtype was associated with poorer survival outcomes (HR = 1.79, 95%CI: 1.17–2.74, p = 0.0062, Fig. 2A). Next, we replicated the effect of the ATII-3 cell subtype on survival outcomes in GSE27957 and GSE28042, we found that a high proportion of the ATII-3 cell subtype was also associated with poorer survival outcomes (in GSE27957, HR = 4.46, 95%CI: 1.39–14.3, p = 0.0064, Fig. 2B and in GSE28042, HR = 2.33, 95%CI: 1.13–4.77, p = 0.018, Fig. 2C). These findings indicated that ATII-3 was a risk cell subtype associated with IPF patients’ prognosis.

496 ATII cells were divided into three cell subtypes, namely ATII-1(210 cells, 46%), ATII-2(181 cells, 40%), and ATII-3(64 cells, 14%) (Fig. 1A, C). SFTPC and LAMP3 were typical markers for ATII cells [20], which were upregulated in three ATII cell subtypes (Fig. 2D). Next, we identified the marker genes of three ATII cell subtypes, CCDC141 was upregulated in the ATII-1cell subtype and ATII-2 cell subtype (Fig. 2E), while DMBT1 upregulated in ATII-1 (Fig. 2F). CCL20 was upregulated only in the ATII-3 cell subtype (Fig. 2G). As ATII-3 was a risk cell subtype associated with IPF patients’ prognosis, we named ATII-3 as ATII-CCL20 to indicate its specificity in the following research. CCL20 (C–C motif chemokine ligand 20) acted as a ligand for C–C chemokine receptor CCR6 and was expressed by epithelial cells in various diseases [54, 55]. CCL20 was associated with the TGF-β pathway, which played a key role in fibrosis formation and inflammation development [56].

To confirm the repeatability and reliability of the ATII-CCL20 cell subtype, we performed clustering on 24,891 epithelial cells in the validation dataset, GSE135893. The results indicated that ATII cells were classified into 5 cell subtypes, with a cell subtype (495 cells, constituting 13% of the ATII cells) showing high expression of CCL20 (Additional file 1: Figure S3). This finding is highly consistent with the results obtained from GSE136831. It suggests that the identification of the ATII-CCL20 cell subtype is not a random occurrence.

### Biological characteristics of ATII-CCL20 cell subtype in single cell

**Fig. 3:**
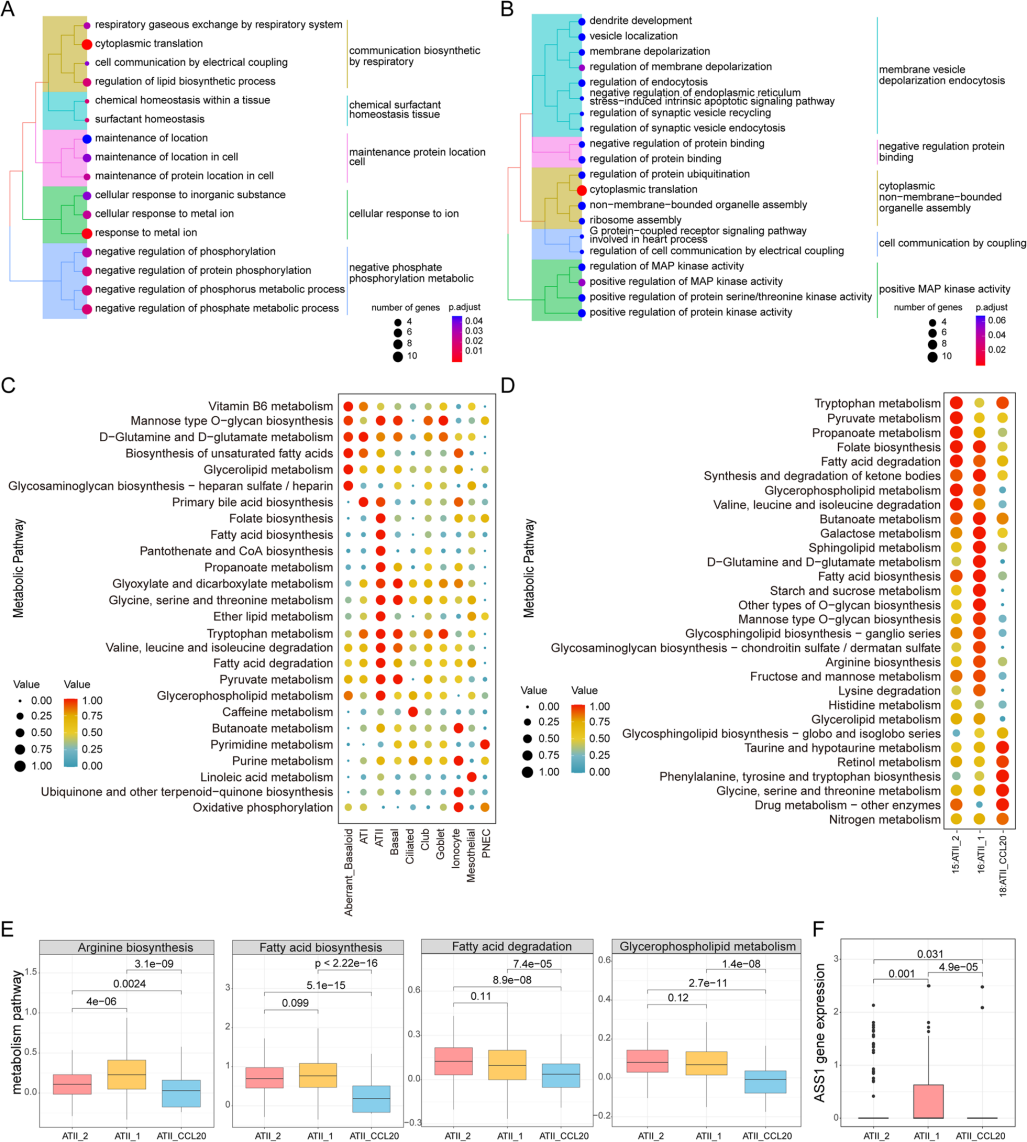
Different biological characteristics between ATII-CCL20 cell subtype and ATII-1, ATII-2. **A**, **B** Enrichment analysis of DEGs between ATII-CCL20 and ATII-1 cell subtype (**A**) and ATII-2 cell subtype (**B**). **C**, **D** Dot plots displayed metabolic pathways scores across 10 major epithelial cell types (**C**), ATII-CCL20 cell subtype and ATII-1, ATII-2 cell subtype (**D**). **E**, **F** A box plot showed differences in lipid metabolism scores (**E**) and ASS1 gene expression (**F**) between the ATII-CCL20 cell subtype and ATII-1, ATII-2 cell subtype

To further investigate the differences in biological characteristics between the ATII-CCL20 cell subtype and ATII-1, ATII-2 cell subtype, we performed differential analysis. 88 DEGs were identified between ATII-CCL20 and ATII-1 cell subtypes based on a defined threshold, and these genes were significantly enriched in negative phosphate phosphorylation metabolic, cellular response to ion, maintenance protein location cell, chemical surfactant homeostasis tissue, and communication biosynthetic by respiratory (Fig. 3A). While 49 DEGs were identified between ATII-CCL20 and ATII-2 cell subtypes, these genes were significantly enriched in positive MAP kinase activity, cell communication by coupling, cytoplasmic non-membrane-bounded organelle assembly, negative regulation protein binding, and membrane vesicle depolarization endocytosis (Fig. 3B). These results indicated that there was obvious heterogeneity in biological characteristics between the three ATII cell subtypes.

Studies have shown that IPF was usually accompanied by metabolic disorders of carbohydrates, lipids, proteins, and hormones, which might provide a new strategy for treating IPF [57]. Therefore, we conducted a metabolic analysis. Firstly, by evaluating the scores of different metabolic pathways among 10 major cell types, it was found that ATII cells were associated with some lipid metabolism pathways, such as glycerophospholipid metabolism, fatty acid degradation, fatty acid biosynthesis, and ether lipid metabolism (Fig. 3C). Secondly, the scores of different metabolic pathways were analyzed in the ATII-CCL20 cell subtype relative to ATII-1 and ATII-2, and it was found that the metabolic score of ATII-CCL20 cell subtype was lower in sphingolipid metabolism, glycerophospholipid metabolism, galactose metabolism, and fructose and mannose metabolism (Fig. 3D). Particularly, in the functions related to arginine biosynthesis, fatty acid biosynthesis, fatty acid degradation, and glycerophospholipid metabolism (p < 0.05, Fig. 3E), the metabolic pathways scores of the aforementioned functions in ATII-CCL20 cell subtype were lower than those in ATII-1 and ATII-2. This indicated that many metabolic pathways in the ATII-CCL20 cell subtype might be depleted. The arginine biosynthesis has the highest metabolic score in ATII-1, a moderate score in ATII-2, and the lowest score in ATII-CCL20, even lower than the average metabolic score of arginine biosynthesis in ATII cells. Arginosuccinate synthase 1 (ASS1), a key enzyme in arginine biosynthesis [58], we found that ASS1 exhibits the lowest expression level in the ATII-CCL20 cell subtype. These findings suggest that the ATII-CCL20 cell subtype may influence arginine biosynthesis by downregulating ASS1 in IPF patients (p < 0.05, Fig. 3F). Studies had shown that lipid metabolism was a special metabolic pathway in the lungs, mainly utilizing fatty acid oxidation for energy supply under hypoxic conditions [59]. Triglycerides, phospholipids, and sphingolipids were important components of the human body and were also important components of surfactants synthesized by alveoli cells, playing an important role in maintaining normal alveolar surface tension [29, 60, 61]. Dysregulation of lipid metabolism in IPF not only reduced the repair function of AT2 cells but also promoted the transformation of fibroblasts to myofibroblasts [62]. Therefore, our study not only confirmed the critical role of lipid metabolism in IPF but also identified the cell subtype ATII-CCL20 that was closely related to lipid metabolism processes in IPF, which might be helpful for future treatment of IPF.

**Fig. 4:**
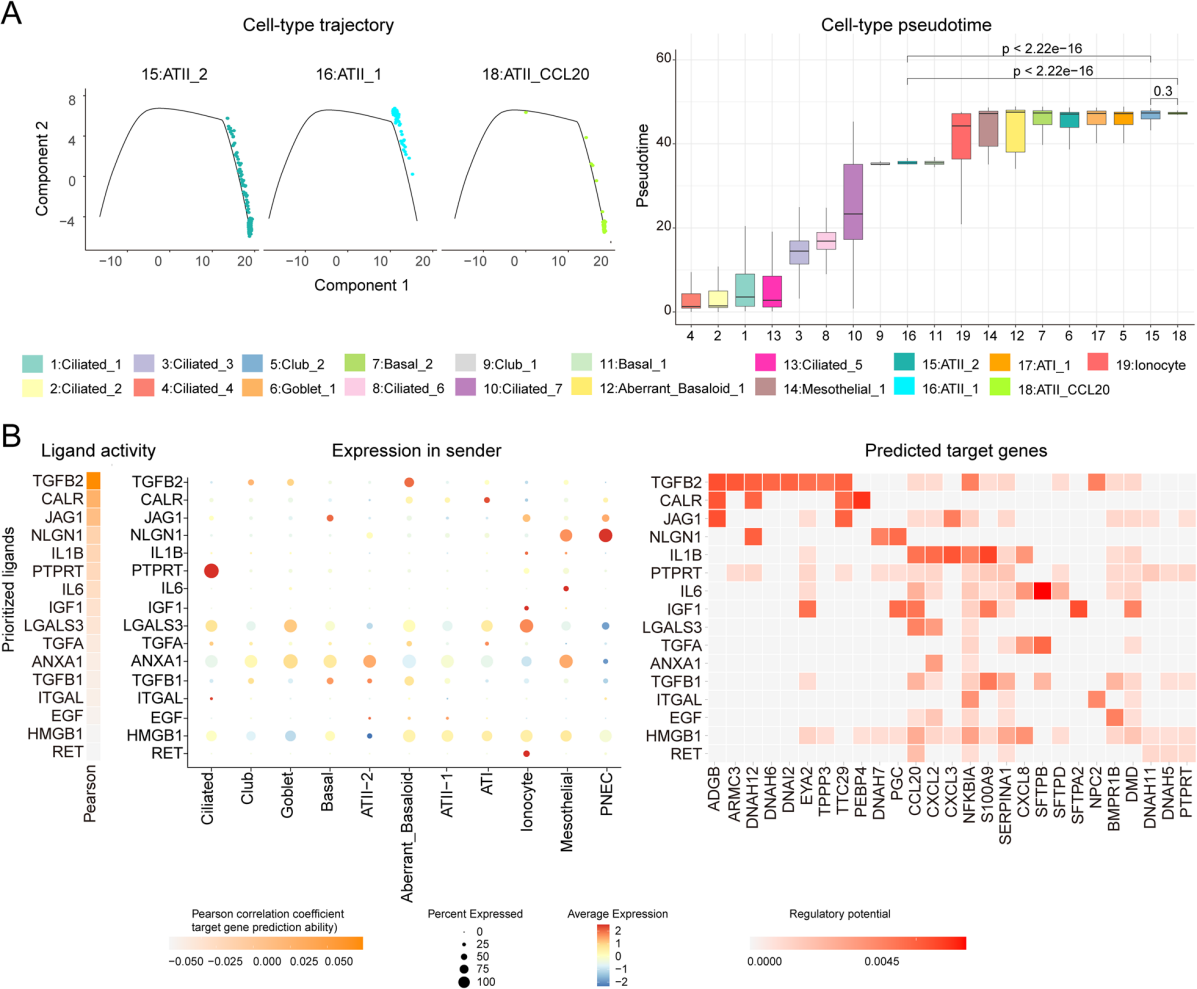
ATII-CCL20 cell subtype was regulated by other epithelial cells. **A** Pseudo-temporal trajectory and box plot showed the high differentiation level of the ATII-CCL20 cell subtype. **B** Intercellular communication analysis indicated that the ATII-CCL20 cell subtype was controlled by other epithelial cells

To reveal the developmental process of the ATII-CCL20 cell subtype relative to the ATII-1 cell subtype and ATII-2 cell subtype, R package Monocle was used for pseudo-time-based cell trajectory inference analysis of all epithelial cells. It was noteworthy that the pseudotime distribution of the ATII-CCL20 cell subtype among all epithelial cells was in the last stage, indicating that the ATII-CCL20 cell subtype may be the final state of epithelial cell differentiation in IPF patients. Meanwhile, the ATII-1 cell subtype was closely adjacent to the ATII-CCL20 cell subtype, meaning that the ATII-1 cell subtype was most likely to transform into the ATII-CCL20 cell subtype (Fig. 4A). Next, to analyze the intercellular communication between ATII-CCL20 and other epithelial cell subtypes, R package NicheNet was used to analyze the regulatory effects of ligands from the other 9 major cell types as well as ATII-1 and ATII-2 on ATII-CCL20 cell subtype. It was found that TGFB2 derived from abnormal stromal cells, PTPRT derived from PNEC cells, and NLGN1 derived from Ciliated cells, exhibited strong regulatory effects on the ATII-CCL20 cell subtype. TGFB2 simultaneously regulated receptors such as ADGB, ARMC3, and DNAH12. Therefore, it was speculated that the ATII-CCL20 cell subtype was regulated by abnormal stromal cells through its receptor expressing TGFB2, leading to functional changes and affecting the prognosis of IPF patients (Fig. 4B).

### Comprehensive functional analysis among ATII-CCL20 groups using IPF bulk RNA-seq data

**Fig. 5:**
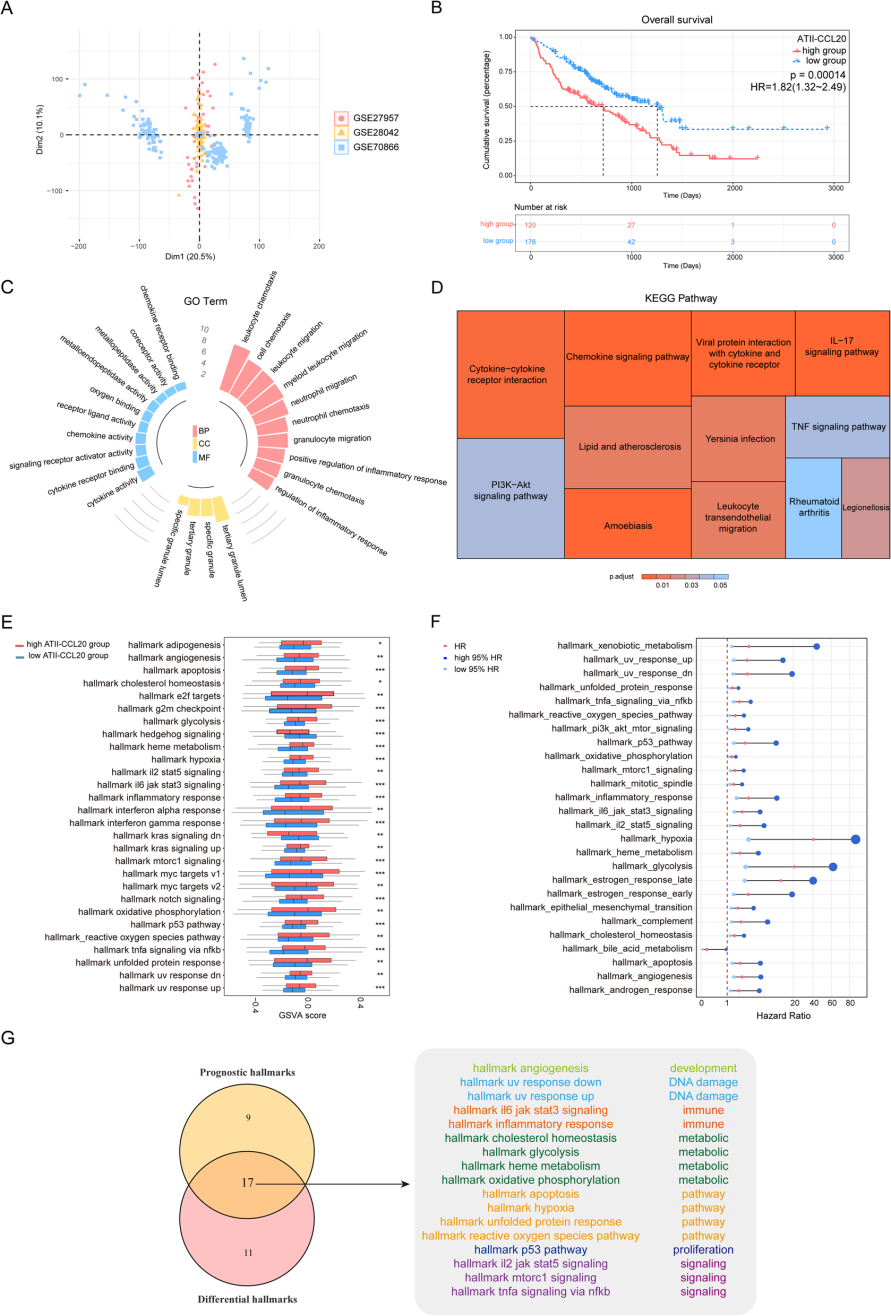
IPF patients with high ATII-CCL20 proportion in integrated data had a worse prognosis. **A** GSE27957, GSE28042, and GSE70866 data integration and batch effect removal were performed for the subsequent analysis. **B** The difference in OS between IPF patients with high and low ATII-CCL20 proportion in the integrated data. **C**, **D** GO enrichment analysis and KEGG pathway enrichment analysis of DEGs between IPF patients with high and low ATII-CCL20 proportion in the integrated data. **E** Box plots showed the different GSVA scores of hallmark pathways in IPF patients with high and low ATII-CCL20 proportion, *p < 0.05, **p < 0.01, ***p < 0.001. **F** Univariate Cox analysis revealed the correlation between the GSVA score of hallmark pathways and OS in IPF patients. **G** A Venn diagram showed 17 hallmark pathways that were useful for risk stratification of IPF patients based on ATII-CCL20 proportion

To explore the biological functions of IPF patients with high ATII-CCL20 cell subtype proportion, we merged the three datasets and removed batch effects (Fig. 5A). Based on the ATII-CCL20 proportion, 120 IPF patients were classified as a high-proportion group and 176 IPF patients were classified as a low-proportion group. The results showed that IPF patients with a higher proportion of ATII-CCL20 had a worse prognosis (HR = 1.82, p = 0.00014, Fig. 5B). Next, differential analysis was performed on the two groups of IPF patients, and 298 DEGs were identified based on a defined threshold, including 61 upregulated DEGs and 237 downregulated DEGs. These DEGs mainly regulated inflammatory response-related functions such as leukocyte chemotaxis, leukocyte migration, myeloid leukocyte migration, and positive regulation of inflammatory response (Fig. 5C). KEGG pathway enrichment analysis of the DEGs showed that they were enriched in immune-related pathways such as the chemokine signaling pathway, IL-17 signaling pathway, cytokine-cytokine receptor interaction, and leukocyte transendothelial migration. Additionally, the DEGs were closely associated with rheumatoid arthritis, amoebiasis, and bacterial infections such as Legionellosis, and Yersinia (Fig. 5D). These DEGs were also enriched in the PI3K-Akt signaling pathway and TNF signaling pathway. Studies have shown that the PI3K-Akt signaling pathway was directly involved in the formation of IPF or cooperated with other pathways to promote the development of IPF [63], while the TNF signaling pathway was upregulated in bleomycin-induced fibrotic lung tissue and TNF-α-induced NF-κB activation promoted fibroblast differentiation and exacerbated bleomycin-induced pulmonary fibrosis [64]. These results indicated that the IPF patients with a high proportion of ATII-CCL20 mainly activated immune-related functions to promote the formation of IPF, resulting in poor prognosis.

To further explore the hallmark pathways associated with ATII-CCL20 proportion, GSVA was performed on IPF patients with a high and low proportion of ATII-CCL20. Compared to IPF patients with a low proportion of ATII-CCL20, the GSVA scores of 26 hallmark pathways were increased in IPF patients with a high proportion of ATII-CCL20 (p < 0.05, Fig. 5E). Kaplan–Meier analysis was used to evaluate the prognostic impact of 50 hallmark pathways, and the results showed that 25 hallmark pathways were risk factors for the prognosis of IPF patients (HR > 1, p < 0.05, Fig. 5F). The intersection of dysregulated hallmark pathways and risk hallmark pathways yielded 17 hallmark pathways related to ATII-CCL20 proportion, mainly affecting immune, metabolic, DNA damage, and multiple signaling pathways (Fig. 5G). The GSVA scores of 17 hallmark pathways were calculated in IPF patients with high and low ATII-CCL20 proportions in GSE47460. The results showed that out of 17 hallmark pathways, 10 were correlated with the ATII-CCL20 proportion in lung tissues (Additional file 1: Figure S4A).

**Fig. 6:**
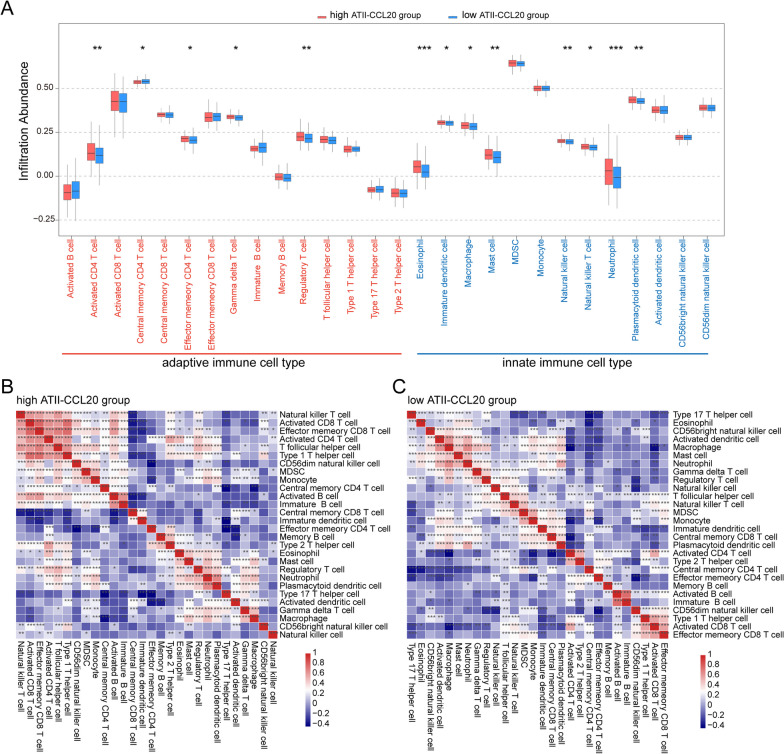
ATII-CCL20 proportion was associated with immune cell infiltration estimated by ssGSEA. **A** Differences of immune cell infiltration between IPF patients with high and low ATII-CCL20 proportion. **B**, **C** Correlations between immune cell infiltration in IPF patients with high (**B**) and low (**C**) ATII-CCL20 proportion, respectively. *p < 0.05, **p < 0.01, ***p < 0.001

Next, we explored the differences in immune cell content between IPF patients with a high and low proportion of ATII-CCL20. The box plots showed that the infiltrating immune cells inferred by the ssGSEA algorithm were more immune cells infiltrated in IPF patients with a high proportion of ATII-CCL20 (p < 0.05, Fig. 6A). Among them, there were five types of adaptive immune cells, including activated CD4 T cells, regulatory T cells, etc. (red), which were correlated with high ATII-CCL20 proportion, while innate immune cell types such as mast cells, eosinophils, neutrophils, etc. (blue) were correlated with high ATII-CCL20 proportion. In addition, the correlation between infiltrating immune cells in the two groups of IPF patients was analyzed separately. The results showed that the correlations between infiltrating immune cells in IPF patients with a high proportion of ATII-CCL20 were stronger, such as positive correlations between natural killer T cells, activated CD8 T cells, effector memory CD8 T cells, and activated CD4 T cells (Fig. 6B), while the correlations between infiltrating immune cells in IPF patients with a low proportion of ATII-CCL20 was weaker (Fig. 6C). These findings strongly suggested that there were differential immune statuses among ATII-CCL20 groups and the infiltration of immune cells played a crucial role in the risk stratification of IPF patients. At the same time, we found that the lung tissue samples in GSE47460, IPF patients with a high ATII-CCL20 proportion also exhibited higher levels of immune cell infiltration (Additional file 1: Figure S4B).

### Establishment of the prognostic nomogram

**Fig. 7:**
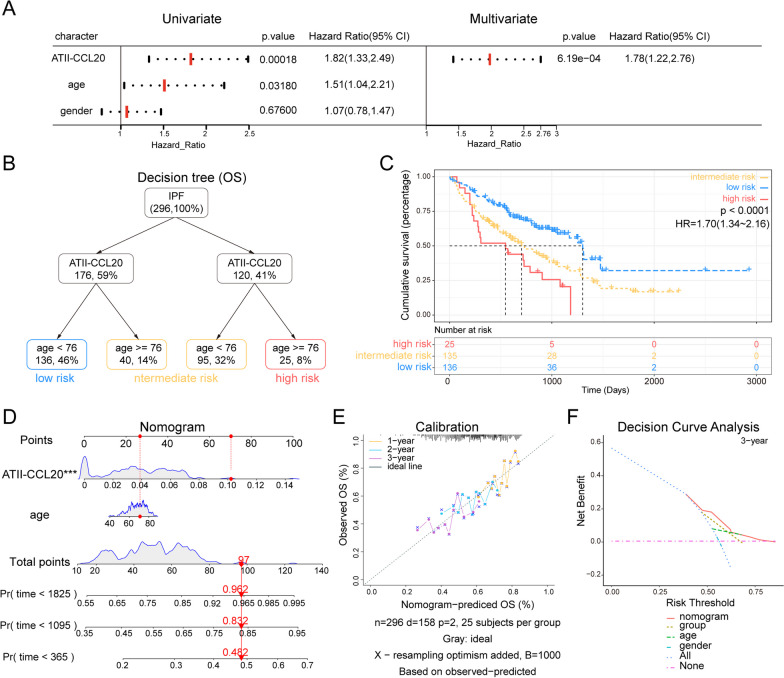
Generated survival decision tree and nomogram to improve risk stratification and estimate survival probabilities of IPF patients. **A** Univariate and multivariate COX analysis of clinical characteristics and ATII-CCL20 proportion. **B** Construction of a survival decision tree using age and ATII-CCL20 proportion to optimize risk stratification. **C** The Kaplan–Meier survival curves of OS in three risk subgroups. **D** Details of the nomogram were used to predict the probability of 1-year, 3-year, and 5-year OS of IPF patients. **E** The calibration plot showed high accuracy of the nomogram. **F** Decision curve analysis showed that the nomogram had the best survival prediction ability compared to other clinical characteristics

To determine whether ATII-CCL20 proportion was an independent indicator of prognosis in IPF patients, 296 IPF patients were selected, whose clinical annotations included gender and age. Univariate and multivariate COX analyses were performed with three variables, including ATII-CCL20 proportion, gender, and age. In both univariate and multivariate COX analyses, the HR for ATII-CCL20 proportion were 1.82 (95% CI: 1.33–2.49) and 1.78 (95% CI: 1.22–2.76), respectively, higher than that for gender and age (Fig. 7A). Importantly, the multivariate analysis showed that the ATII-CCL20 proportion was an independent prognostic factor for IPF.

In the univariate COX analysis, we found that both age and ATII-CCL20 proportion affected the survival outcomes of IPF patients. Therefore, recursive partitioning analysis was performed using ATII-CCL20 proportion and age to construct a survival decision tree and optimize the risk stratification of IPF patients. As shown in the decision tree (Fig. 7B), three different risk subgroups were defined based on two main components, including ATII-CCL20 proportion and age (76 years old as the cut-off point for age). IPF patients with low ATII-CCL20 proportion and age < 76 were defined as the “low-risk” group, while those with high ATII-CCL20 proportion and age > 76 were labeled as the “high-risk” group. The remaining patients were defined as “intermediate-risk” patients. A significant difference in OS was observed among the three risk subgroups (HR = 1.7, p < 0.0001, Fig. 7C). To quantify the risk assessment of IPF patients, a nomogram was generated using ATII-CCL20 proportion and age, and an example was shown by the red arrow (Fig. 7D). In the calibration analysis, the predicted lines of the nomogram for 2-year and 3-year survival probability were very close to ideal performance (Fig. 7E), indicating that the nomogram had high accuracy. Compared with other clinicopathological characteristics, the nomogram showed the strongest predictive ability for OS in IPF patients (Fig. 7F).

## Discussion

IPF is a rare fibrotic lung disease with a poor prognosis and different clinical progression. Genetic studies have shown that changes in lung epithelial cells were the basis for the occurrence and development of IPF [65, 66]. Therefore, exploring the heterogeneity of epithelial cell types would provide a new direction for studying IPF. Therefore, exploring the heterogeneity of epithelial cell types would provide a new direction for studying IPF. In this study, we utilized an integrated transcriptomic framework incorporating bulk RNA-seq data and scRNA-seq data from IPF patients. Through this approach, we identified a cell subtype, ATII-CCL20, that is associated with poor survival outcomes in IPF patients. Moreover, we found that the lipid metabolism level in ATII-CCL20 was significantly lower than that in other ATII cells, and ATII-CCL20 was a highly differentiated epithelial cell subtype. The IPF patients with a high proportion of ATII-CCL20 were more likely to experience inflammatory reactions and metabolic disorders. We constructed a prognosis model based on the proportion of ATII-CCL20 and clinical indicators to predict the survival time of IPF patients, which had significant implications for personalized management.

Alveolar epithelial cells (AECs), as key cells maintaining the structure and function of the lung, were extremely important in the development of IPF [67]. AECs had two types: alveolar type I cells (ATI) and alveolar type II cells (ATII) [68]. Physiologically, ATII cells could proliferate and differentiate into ATI cells. The proliferation, differentiation, and apoptosis of ATII cells were in a dynamic equilibrium to maintain the normal structure and function of the alveolar epithelium [68, 69]. Under conditions of repeated and sustained injury stimuli, ATII cells underwent damage and repair, resulting in the secretion of various pro-fibrotic cytokines, further inducing fibroblast proliferation and differentiation into highly active myofibroblasts with the ability to synthesize ECM [19, 70, 71]. Excessive deposition of ECM ultimately led to the deformation and destruction of the alveolar structure [67]. Therefore, ATII cells were key components of IPF initiation and progression. However, whether the changes in ATII cells were related to the prognosis of IPF patients and their related mechanisms were not yet clear. Our study showed that there was indeed a special ATII cell subtype (named ATII-CCL20), and the higher proportion of ATII-CCL20 cell subtype in IPF patients was associated with poorer prognosis. We analyzed in detail the potential mechanisms by which the ATII-CCL20 cell subtype affected the prognosis of IPF patients, and found that the ATII-CCL20 cell subtype was closely associated with metabolic dysfunction, both at the single-cell level and at the individual patient level. Compared with other ATII cells, the lipid metabolism function of the ATII-CCL20 cell subtype was downregulated. The higher the proportion of the ATII-CCL20 cell subtype, the more pronounced the lipid metabolism abnormalities, and the more severe the fibrosis and poorer prognosis in IPF patients. However, the potential mechanism by which ATII-CCL20 affected the prognosis of IPF patients based on genetic analysis remains to be validated through relevant biological experiments for accuracy. Finally, considering the clinical applications, we constructed a prognosis model for IPF patients based on the proportion of ATII-CCL20 and patients’ clinical indicators to address practical clinical problems, which had significant implications.

Research indicates that the progression of idiopathic pulmonary fibrosis (IPF) is associated with severe lung injury, leading to the accumulation of a large number of macrophages. These macrophages, through the production of various cytokines, trigger inflammatory responses [72]. Additionally, in mouse models, it has been demonstrated that targeting proteins in macrophages can improve the condition of IPF [73–76], highlighting the potential of macrophages as a therapeutic target for pulmonary fibrosis. In our study, we observed a higher presence of macrophages in IPF patients with elevated levels of ATII-CCL20, suggesting that the increased abundance of macrophages may be a characteristic closely associated with disease progression and inflammatory responses in individuals with high ATII-CCL20 levels.

Given the poor survival outcomes of patients diagnosed with IPF, it is essential to further understand the factors that affect IPF survival outcomes. Currently, clinical prognostic tools for IPF mainly rely on the patient’s GAP (gender, age, and physiology) index [77]. We demonstrated that evaluating the cell composition of patients might predict the IPF patients’ prognosis, the high proportion of ATII-CCL20 cell subtype associated with poor prognosis. The rare ATII-CCL20 cell subtype may potentially become a therapeutic target in the future, highlighting the need for more in-depth research to improve current clinical treatment strategies. Meanwhile, our results showed that the proportion of ATII-CCL20 cell subtype in PBMC data was significantly associated with the prognosis of IPF patients, which indicated the potential of liquid biopsy to infer the IPF patients’ prognosis. In the end, we compared the BALF and PBMC data with the lung tissue data. BALF and PBMC data contained relatively few epithelial cells, whereas lung tissue data contained a large number of epithelial cells. However, the biological differences between patients with different proportions of ATII-CCL20 in BALF and PBMC datasets were highly consistent with the biological differences between samples with different proportions of ATII-CCL20 in lung tissue. This suggests that the identified risk cell subtypes in the BALF and PBMC datasets are reliable.

Research has shown that the risk of developing lung cancer in patients with IPF is nearly five times higher compared to the general population [78, 79]. It is noteworthy that CCL20 exhibits a promotive role in tumor development, specifically in lung adenocarcinoma [80], by promoting the epithelial-mesenchymal transition process. At the same time, the high expression of CCL20 in LUAD patients is closely associated with poor prognosis [81]. These results indicate that a high level of ATII-CCL20 cell subtype may contribute to the occurrence and poor prognosis of IPF-related lung cancer. Simultaneously, PD-1 and PD-L1 inhibitors have achieved significant success in cancer treatment [82, 83]. Some studies indicate that Pembrolizumab can alleviate bleomycin-induced lung fibrosis [84]. This suggests a potential benefit of anti-PD-1 and anti-PD-L1 treatments in alleviating IPF. Nevertheless, further research is needed to confirm their effectiveness and safety.

Although this study improved our understanding of an alveolar type II cell subtype that was associated with the IPF patients’ prognosis, it had limitations. Firstly, this study revealed the association between ATII-CCL20 with survival outcomes in multiple IPF datasets and constructed a nomogram model to achieve prognosis prediction. However, some clinically relevant factors (for example, smoking, metal/wood dust inhalation, genetic factors, physiological indicators, and comorbidities) of IPF patients were not explored in our nomogram model, as some of the IPF patients lacked these parameters. Therefore, larger-scale studies were needed to explore the IPF patients’ prognosis in different contexts, especially considering the differences in cell composition. Secondly, this study was based on retrospective data from GEO, and the sample size in each dataset was relatively small. The scRNA-seq data and bulk RNA-seq data in this study were not from the same sample, and the inferred epithelial cell composition of IPF patients based on deconvolution algorithms might differ from the actual situation. So more comprehensive studies based on scRNA-seq and bulk RNA-seq data from the same IPF sample were needed.

### Supplementary Information

**Additional file 1.** **Table S1**. Details of baseline information in 4 public datasets. **Figure S1**. Workflow. **Figure S2.** The composition of 10 major epithelial cell types (A) and 19 independent cell subtypes (B) in 32 IPF patients. **Figure S3.** Analysis of the epithelial cell in IPF patients using scRNA-seq data GSE135893. (A) tSNE plots of major epithelial cell types. (B) tSNE plots of 24 cell subtypes. (C) CCL20 expression level in 24 cell subtypes. **Figure S4.** The comprehensive functional analysis among ATII-CCL20 groups in GSE47460. (A) GSVA scores of hallmark pathways in IPF patients with high and low ATII-CCL20 proportion. (B) Differences of immune cell infiltration between IPF patients with high and low ATII-CCL20 proportion.

## Data Availability

The data that support the findings of this study are available from GEO, but restrictions apply to the availability of these data, which were used under license for the current study, and so are not publicly available. Data are however available from the authors upon reasonable request and with permission of GEO.
