# Supplementary material for: Identifying a survival-associated cell type based on multi-level transcriptome analysis in idiopathic pulmonary fibrosis
Source: Respir Res. 2024 Mar 15;25:126. doi: 10.1186/s12931-024-02738-w (PMC10941445; doi:10.1186/s12931-024-02738-w)

**Additional Information**

**Table S1** Details of baseline information in 4 public datasets.

|  |  | GSE70866 | GSE27957 | GSE28042 | GSE47460 |
| --- | --- | --- | --- | --- | --- |
| number of IPF patients |  | 176 | 45 | 75 | 160 |
| tissue |  | BALF | PBMC | PBMC | Lung Tissue |
| status | dead | 100 | 15 | 43 |  |
|  | alive | 76 | 30 | 32 |  |
| age | age>=76 | 44 | 6 | 15 | 11 |
|  | age<76 | 132 | 39 | 60 | 149 |
| gender | man | 144 | 40 | 52 | 110 |
|  | woman | 32 | 5 | 23 | 50 |

**Figure S1** Workflow

**
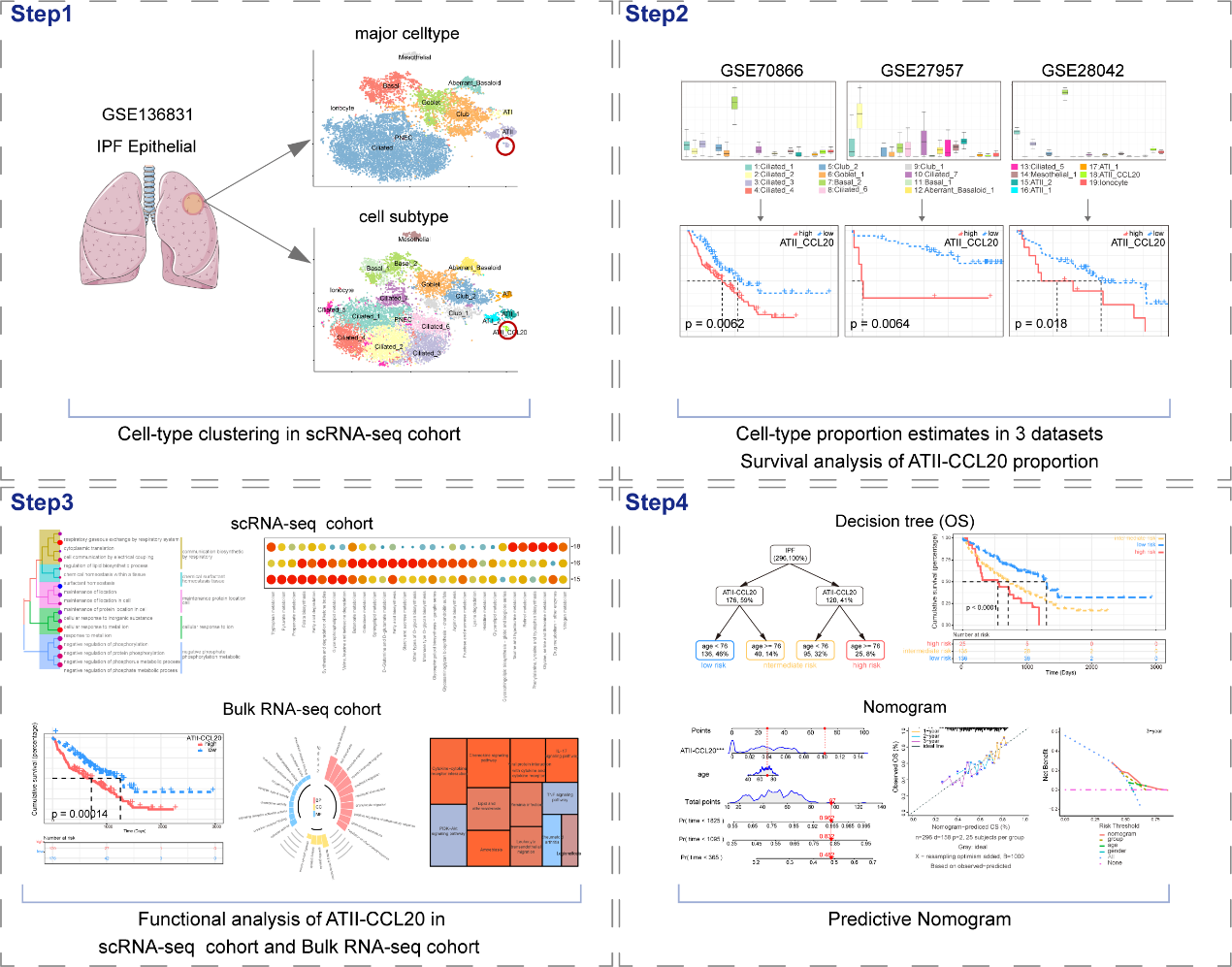
**

**Figure S2.** The composition of 10 major epithelial cell types (A) and 19 independent cell subtypes (B) in 32 IPF patients.


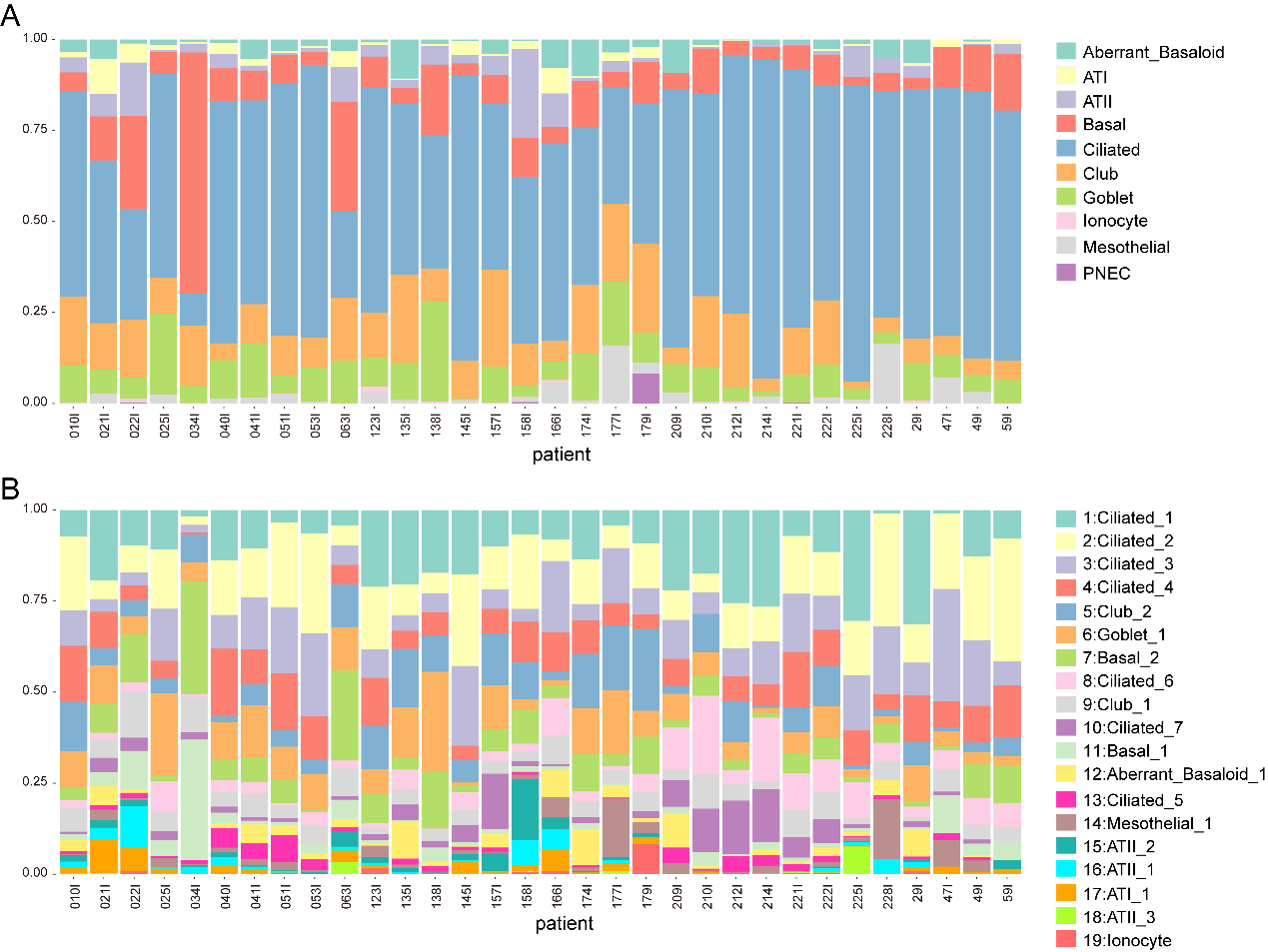


**Figure S3.**

Analysis of the epithelial cell in IPF patients using scRNA-seq data GSE135893. (A) tSNE plots of major epithelial cell types. (B) tSNE plots of 24 cell subtypes. (C) CCL20 expression level in 24 cell subtypes.


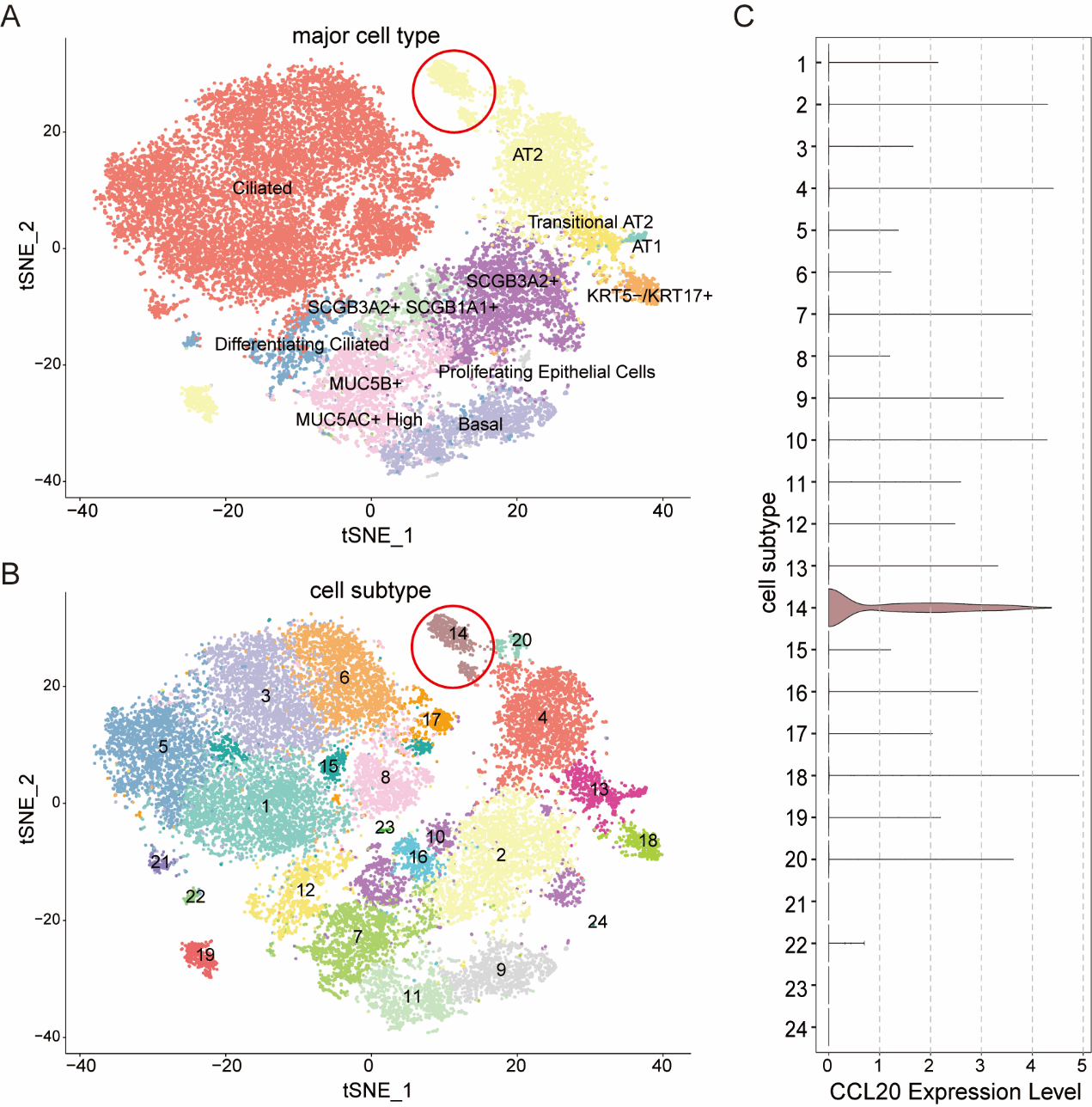


**Figure S4.** The comprehensive functional analysis among ATII-CCL20 groups in GSE47460. (A) GSVA scores of hallmark pathways in IPF patients with high and low ATII-CCL20 proportion. (B) Differences of immune cell infiltration between IPF patients with high and low ATII-CCL20 proportion.


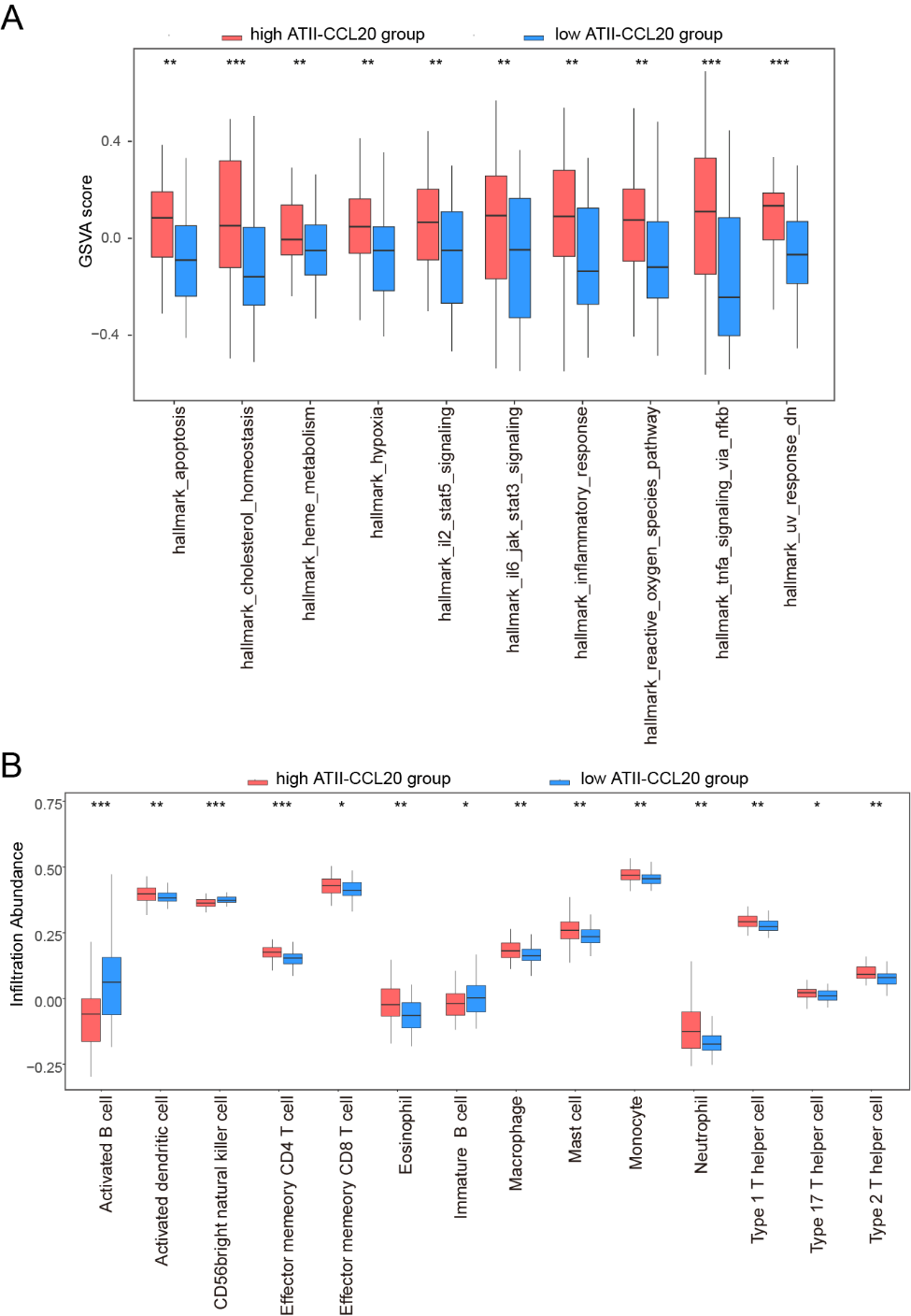

Supplement: Supplementary file 1 — Additional file 1. Table S1. Details of baseline information in 4 public datasets. Figure S1. Workflow. Figure S2. The composition of 10 major epithelial cell types (A) and 19 independent cell subtypes (B) in 32 IPF patients. Figure S3. Analysis of the epithelial cell in IPF patients using scRNA-seq data GSE135893. (A) tSNE plots of major epithelial cell types. (B) tSNE plots of 24 cell subtypes. (C) CCL20 expression level in 24 cell subtypes. Figure S4. The comprehensive functional analysis among ATII-CCL20 groups in GSE47460. (A) GSVA scores of hallmark pathways in IPF patients with high and low ATII-CCL20 proportion. (B) Differences of immune cell infiltration between IPF patients with high and low ATII-CCL20 proportion. [file 12931_2024_2738_MOESM1_ESM.docx]
